# Assessment of Hospital Rooming-in Practice in Abu Dhabi, United Arab Emirates: A Cross-Sectional Multi-Center Study

**DOI:** 10.3390/nu12082318

**Published:** 2020-08-01

**Authors:** Zainab Taha, Ahmed Ali Hassan, Ludmilla Wikkeling-Scott, Ruba Eltoum, Dimitrios Papandreou

**Affiliations:** 1 Department of Health Sciences, College of Natural and Health Sciences, Zayed University, Abu Dhabi P.O. Box 144534, UAE; Ludmilla.scott@gmail.com (L.W.-S.); Dimitrios.Papandreou@zu.ac.ae (D.P.); 2 Taami for Agricultural and Animal Production, Khartoum, Sudan; aa801181@gmail.com; 3 Faculty of Medicine, Charles University, 500 03 Hradec Kralove, Czech Republic; eltoumr@lfhk.cuni.cz

**Keywords:** rooming-in, initiation of breastfeeding, gestational age, body mass index, United Arab Emirates

## Abstract

The World Health Organization (WHO) recommends rooming-in to reduce infant mortality rates. Little research has been done to assess practices such as rooming-in and its relation to breastfeeding in the United Arab Emirates (UAE). The aim of this study was to examine the prevalence of rooming-in during hospital stay among mothers with infants six months old and below, in addition to other associated factors in Abu Dhabi, UAE. This study utilized a sub-sample extracted from a dataset based on a convenience sample of mothers who were recruited from governmental maternal and child health centers as well as from the community. The purpose of the original research was to evaluate infant and young children’s feeding practices. A pre-tested questionnaire was used during interviews with mothers once ethical clearance was in place. Multivariable logistic regression was conducted to describe the results. The original sample included 1822 participants, of which 804 infants met the inclusion criteria. The mean age for mothers and infants was 30.3 years and 3.5 months, respectively. The rate of rooming-in during hospital stay was 97.5%. Multivariable logistic regression analysis indicated factors associated with not rooming-in were low maternal age (Adjusted Odds Ratios (AOR) = 1.15, 95% confidence interval (CI): 1.03, 1.30), low gestational age (GA) (AOR = 1.90, 95% CI: 1.52, 2.36), abnormal pre-pregnancy body mass index (BMI) (AOR = 3.77, 95 % CI: 1.22, 11.76), and delayed initiation of breastfeeding (AOR = 4.47, 95 % CI: 1.08, 18.48). In the context of the high rate of rooming-in revealed in this study, there should be a focus on those groups who do not room-in (i.e., younger women and those with babies of a younger gestational age). Rooming-in practice provides self-confidence in taking care of a baby, knowledge about breastfeeding, and stimulates early-phase lactation.

## 1. Introduction

The practice of rooming-in, as recommended by the World Health Organization (WHO) and United Nations Children’s Fund (UNICEF), is one of the ten steps for hospitals with maternity facilities to be accredited as a Baby-Friendly Hospital (BFH) [1]. The practice of rooming-in, as defined by the WHO and UNICEF, is a “hospital practice where postnatal mothers and normal infants stay together in the same room for 24 h a day from the time they arrive in their room after delivery” [2]. The Centers for Disease Control and Prevention (CDC) defines rooming-in as the process in which mothers and their newborns stay together for at least 23 h a day in the hospital [3]. Research shows that rooming-in plays a critical role in initiation of breastfeeding and enhances skin-to-skin contact, thus protecting infants from the risk of infections potentially contracted from other infants or health personnel [4,5]. Other advantages of rooming-in include the promotion of early breastfeeding and encouragement of maternal–infant bonding, increases to the mother’s confidence, and the contribution of protection against stress related to change in the parenting role for some mothers. It has also been well documented that newborn babies kept in a separate nursery, which used to be common practice, would be a reason to receive significantly more breastmilk substitutes and less breastmilk than babies who are kept in the same room with their mothers [6].

Several studies have found a strong association between rooming-in and improved breastfeeding outcomes [7,8]. Research shows the importance of rooming-in stems from birth, whether at home or at a hospital, as the mother’s and infant’s physical and emotional relationship would be established, and their need for each other would continue [9,10]. The more time the mother and newborn spend together, the better the results of breastfeeding [11]. When together, mothers quickly learn their babies’ needs and how best to care for, soothe, and comfort their newborns.

Studies have revealed that mothers who room-in with their babies increase their milk production, breastfeed for longer periods, and are more likely to breastfeed exclusively compared to mothers who have limited contact with their infants (i.e., those with babies in the nursery at night) [11,12,13,14].

A study in the United Arab Emirates (UAE) reported that mothers who kept their infants in the same room after delivery had a successful rate of breastfeeding, which was six times higher than mothers who kept their infants in separate rooms [14]. Previous studies have identified that not rooming-in infants in the mother’s room was amongst the most significant factors associated with not breastfeeding [15,16].

Research has well documented that supporting the quality of hospital-based breastfeeding support services and implementing the ten steps practices will ultimately improve breastfeeding and achieve national and international breastfeeding goals [17,18]. While many studies have been conducted to evaluate breastfeeding practices in the UAE, little has been done to evaluate the practices that are known to improve breastfeeding, such as rooming-in.

Despite the continuous efforts of the programs to improve breastfeeding, there are considerable disparities in breastfeeding outcomes for the UAE, as reported by several studies [19,20]. This study was prompted by the need to assess practices that may influence and encourage breastfeeding practices among mothers in the UAE. Therefore, the aim of this study was to examine the prevalence of and factors associated with rooming-in during hospital stay among mothers with infants aged six months and below in Abu Dhabi, UAE.

## 2. Materials and Methods 

### 2.1. Participants and Data Collection

The study sample included 804 Emirati and non-Emirati mothers with infants aged six months and below. Data were extracted from a larger cross-sectional study where a convenience sample of 1822 mothers was recruited from the community, as well as seven governmental healthcare centers located in different geographical areas in Abu Dhabi from March to September 2017. Mothers with young children attending the centers during the study and from the community were approached by trained bilingual (Arabic and English) female research assistants, who provided oral and written information about the study. Mothers who met the inclusion criteria of the main project, having at least one child under two years of age, were interviewed by the research assistants using a structured questionnaire. The purpose of the original research was to evaluate infant and young children’s feeding practices. The study was approved by the Research Ethics Committee of Zayed University (ZU17_006_F), and all participants provided written informed consent.

### 2.2. Study Instrument

The study instrument was a questionnaire previously validated by conducting a pilot study where the study team utilized face validity prior to distribution. Participants were divided into two categories, namely rooming-in and not rooming-in. To describe results, these two groups were compared based on factors to include: family demographics (e.g., education, age, nationality, occupation), child’s information (e.g., birth weight and length, delivery mode), and infant feeding practices (e.g., initiation of breastfeeding and exclusive breastfeeding). The questionnaire was first designed in English and then interpreted in Arabic, using a cross-translation strategy, where a local Arabic speaker transformed the English document into Arabic. Thereafter, another local Arabic speaker, blinded to the original translation, translated the document back to English. Any interpretation errors recognized were addressed by the investigators to minimize errors and produce the final version of the instrument.

### 2.3. Study Inclusion and Exclusion Criteria

All infants aged six months and younger and their mothers, who completed data regarding the main outcome (rooming-in), were included in this study, regardless of the gestational age (GA). The cut-off age of six months was chosen to limit the mothers’ potential for recall bias. All children included in the study were reportedly born without medical complications and had no medical issues at the time of the interview.

### 2.4. Statistical Analysis

Data were collected and analyzed using the Statistical Package for the Social Sciences (SPSS) [21]. Descriptive and inferential analysis was used to describe results. T-test and Chi-square tests were applied to analyze continuous and categorical variables, respectively. Variables with significant *p*-values (<0.05) in univariate analysis were further analyzed using multivariable logistic analysis with the dependent variable (i.e., rooming-in coded as 0 and not rooming-in coded as 1). Other variables such as age, GA, maternal body mass index (BMI), mode of delivery, occupation, and initiation of breastfeeding were used as independent variables. Odds Ratio (OR) and 95% Confidence Interval (CI) were calculated. *P*-value < 0.05 was considered statistically significant.

### 2.5. Ethics

The original study from which these data were extracted was approved (ZU17_006_F) by the Research Ethics Committee at Zayed University, UAE, and the Abu Dhabi Health Services Company. Informed consent was obtained from the mothers. Privacy and confidentiality were ensured by several measures, such as excluding names and personal identifiers throughout the period of data collection and analysis.

### 2.6. Definitions


**Variable**

**The Definition**
Rooming-inThe mother and infant being placed in the same room immediately after leaving the delivery suite, and in the case of cesarean sections when the mother was able to respond to her infant.Not rooming-in groupBabies who were not in the same room with mother since the delivery time, or those who required separation out of rooming-in in the middle of the course due to poor condition of babies or due to maternal condition.Timely initiation of breastfeedingWhen the infant initiated breastfeeding within one hour of birth.Delayed initiation of breastfeedingWhen the infant initiated breastfeeding more than one hour after birth.Exclusive breastfeedingThe infant being fed only breastmilk without any other oral intake, except medications and vitamins, for the first six months of life. It was calculated based on the last 24 h.Breastfeeding supportSupport and encouragement from family (mother, husband, other relatives, and other non-relatives) on breastfeeding.Breastfeeding advice and/or discussionAny received information, positive or negative things, about breastfeeding before or after delivery.Body Mass Index (BMI)It is defined as the weight in kilograms divided by the square of the height in meters (kg/m2). Based on the index, it was subcategorized to normal between 18.50 and 24.99 and abnormal (underweight <18.5, overweight 25–29.9, and obese ≥30) [22]. In the present study, the weight and height data were based on maternal self-reported information. A recent review has documented the accuracy of self-reported pregnancy-related weight [23].

## 3. Results

**Figure 1:**
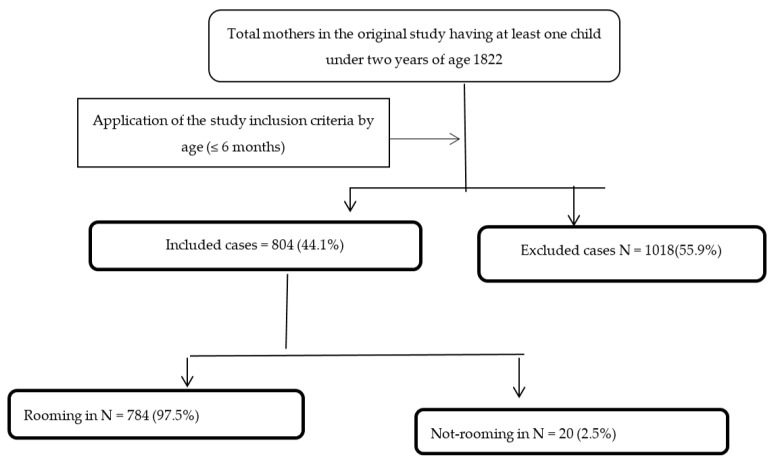
Study participants’ flow chart and main findings.

A total of 804 (44.1%) infants from the original sample (N = 1822) met the inclusion criteria (Figure 1).

First, socio-demographic factors were analyzed to describe their significance among mothers who reportedly roomed-in and those who did not (Table 1). Of the 804 mothers, 784 (97.5%) practiced rooming-in, and 463 (57.6%) initiated breastfeeding within the first hour of delivery. The mean (SD) of maternal age and infants’ age was 30.3 (5.0) years and 3.5 (1.5) months, respectively. The mothers’ ages ranged from 18 to 47 years. The mean and standard deviation (SD) of the infant’s birth weight was 3025 (532.6) g. The mean (SD) of the GA at delivery was 39.2 (1.6) weeks, ranging from 30 to 43 weeks. The pre-term (GA <37 weeks) rate was (5.9%).

One-third of the mothers (N = 265; 33.0%) had abnormal BMI, with 15 (5.7%) of mothers classified as underweight (BMI of <18.5), 209 (78.9%) of mothers classified as overweight (BMI of ≥25 to 29.9), and 41 (15.4%) of mothers classified as obese (BMI of ≥30).

From the 804 mothers, 784 (97.5%) practiced rooming-in, and 463 (57.6%) initiated breastfeeding within the first hour of delivery.

Maternal nationality, having received breastfeeding support, and practicing exclusive breastfeeding, were factors associated with not rooming-in in bivariate analysis only (Table 1).

After establishing the significance of socio-demographic variables and their association with rooming-in, multivariable logistic regression analysis was performed to describe the odds of rooming-in.

Factors associated with not rooming-in were low maternal age AOR = 1.15, 95% CI: 1.03, 1.30, low GA (AOR = 1.90, 95% CI: 1.52, 2.36), abnormal pre-pregnancy BMI (AOR = 3.77, 95% CI: 1.22, 11.76), and delayed initiation of breastfeeding (AOR = 4.47, 95% CI: 1.08, 18.48), which are depicted in Table 2.

## 4. Discussion

Although several studies have examined infant feeding practices in Middle Eastern populations, very few studies have focused specifically on the role of rooming-in [14,24]. Although the Ministry of Health for the UAE has adopted many of the WHO recommendations, included in their implementation of infant feeding policies, breastfeeding practices are sub-optimal. The Federal National Council passed legislation to recommend breastfeeding for the first two years of an infant’s life [19]. As one of the ten steps for a BFH as recommended by WHO, the factor of rooming-in has not been widely studied in the Middle East, and only two Emirates were previously examined in the UAE [14].

The current study contributes to the gap in research in the UAE and highlights the rate of rooming-in practices among mothers and infants in Abu Dhabi. Findings show that a large number of mothers practiced rooming-in (97.5%). These results reflect better practices of rooming-in, as compared to a previous study conducted in the UAE (87.2%) [14]. Similar results of the high rate of rooming-in have been reported in other countries such as South Korea (90.3%) [5]. These rates are high, considering the results of the original study that reported only half of mothers delivered in a BFH [20].

Additional research is necessary to determine whether this pattern is consistent across all seven Emirates, considering that the diversity of the population changes based on geographical location.

Results of this study showed that factors associated with not rooming-in included maternal age, gestational age (GA), pre-pregnancy body mass index (BMI), and initiation of breastfeeding. This study supports other research that has indicated not rooming-in to be associated with low maternal age, which ultimately affects breastfeeding practices [25,26]. Another factor considered in this study relates to maternal physical condition, as measured by BMI. Not rooming-in was more likely among mothers with abnormal BMI. Research has shown that this further reduces the likelihood of breastfeeding practices and breastfeeding duration, which is the ultimate goal for improving infant health [27,28]. Abnormal body weight increases the likelihood of mother–child separation due to pre-term labor and other complications [29,30].

Low GA was significantly associated with not rooming-in, and, even after nursery discharge, pre-term infants are at risk of poor breastfeeding practices such as early weaning [31]. The delay in breastfeeding initiation was also found to be significantly associated with not rooming-in and supports previous findings [15,16]. The previous studies have shown that not rooming-in can be associated with poor breastfeeding practices such as shorter duration of breastfeeding [32]. Therefore, hospitals should always implement rooming-in practice and encourage mothers to initiate and maintain breastfeeding.

One of several factors previously reported was included in this study, namely, cesarean section. Other factors that have been reported in other studies are fatigue [33], lack of support from healthcare providers, perception that routine separation is necessary for bathing, examinations, observation, and other medical procedures [34,35], and cesarean section [33] related difficulties. The gap in research on additional factors that may shed light on rooming-in practices is further highlighted by the current study.

Several limitations should be considered when interpreting the study results. Data for this study represents one of the seven Emirates in the UAE. Although Abu Dhabi is one of the more densely populated Emirates, geographical differences may exist when considering all seven Emirates. Therefore, results are not generalizable to the entire UAE, and additional research is needed. Another limitation in this study is recall bias. Recall bias is common among subjects interviewed about past events [36]. Many studies of mothers and hospitals on breastfeeding practices have relied on data reported retrospectively [37,38]. While prospective designs are preferable, they may not be feasible in some settings. Therefore, the cut-off age of six months was chosen in the current study to limit mothers’ potential for recall bias. In addition, the use of a subsample where the primary researchers did not collect information on maternal health or hospitalization would make it difficult to interpret the results as related to the explanation of the high association between not rooming-in and abnormal BMI. Moreover, the small sample size of the rooming-in group (n = 20) may affect the reliability of the results in detecting any significance, especially in the abnormal BMI group composed of only 11 subjects.

## 5. Conclusions

In the context of the high rate of rooming-in detected in this study, there should be a focus on those groups who do not room-in, such as younger mothers and those with younger gestational-aged babies. Rooming-in promotes both breastfeeding initiation and continuation. Results from this study should serve as the foundation for examining the effects of rooming-in on breastfeeding practices and infant health. In addition, future research is needed to further understand the factors associated with the implementation of rooming-in, as one of the ten steps for a BFH as recommended by WHO. A larger sample size covering subjects from all Emirates would ensure that the sample is considered representative of the UAE and, hence, will support generalization of the results.

## Figures and Tables

**Table 1:** Socio-demographic characteristics of the studied participants in Abu Dhabi, UAE.

	Total (N = 804)		Rooming-in (*n* = 784)		Not Rooming-in (*n* = 20)		*p*-Value
Variables	Mean (SD)		Mean (SD)		Mean (SD)		
Maternal age (years)	30.3 (5.0)		30.4 (5.0)		27.9 (5.4)		0.031
Child age (months)	3.5 (1.5)		3.5 (1.5)		3.9 (1.6)		0.184
Gestational age (weeks)	39.2 (1.6)		39.3 (1.5)		36.0 (3.0)		<0.001
Birth weight (grams)	3025.3(532.6)		3030.5 (517.1)		2822 (960.8)		0.084
Child gender	N	%	N	%	N	%	*p*-value
Female	433	53.9	425	54.2	<10	40	0.208
Male	371	46.1	359	45.8	12	60	
Nationality							
Arab	523	65	279	35.6	<10	10	0.018
Non-Arab	281	35	505	64.4	18	90	
Mode of delivery							
Vaginal delivery	547	68	536	68.4	11	55	0.206
Caesarean section	257	32	248	31.6	<10	45	
Marital Status							
Married	798	99.3	778	99.2	20	100	0.695
Unmarried	<10	0.7	<10	0.8	0	0	
Received breastfeeding support							
Yes	748	93	733	93.5	15	75.0	0.001
No	56	7	51	6.5	<10	25	
Received breastfeeding advice							
Yes	728	90.5	712	90.8	16	80	0.103
No	76	9.5	72	9.2	<10	20	
Pre-pregnancy BMI							
Normal (18.5, 24.9)	539	67.0	530	67.6	<10	45.0	0.034
Abnormal (<18.5 or ≥25)	265	33.0	254	32.4	11	55.0	
Mother’s education							
<Secondary level	22	2.7	22	2.8	0	0	0.447
≥Secondary level	782	97.3	762	97.2	20	100	
Father’s education							
<Secondary level	14	1.7	13	1.7	<10	5	0.259
≥Secondary level	790	98.3	771	98.3	19	95	
Mother’s occupation							
Housewives	522	64.9	512	65.3	10	50.0	0.157
Employed	282	35.1	272	34.7	10	50.0	
Exclusive breastfeeding							
Yes	344	42.8	341	43.5	<10	15	0.011
No	460	57.2	443	56.5	17	85	
Initiation of breastfeeding							
Timely initiated	463	57.6	457	58.3	<10	30.0	0.011
Delayed initiated	341	42.4	327	41.7	14	70.0	
Family income rating							
Good and above	766	95.3	748	95.4	18	90	0.243
Less than good	38	4.7	36	4.6	<10	10	

**Table 2:** Multivariable logistic regression analyses of factors associated with not rooming-in among mothers with infants of six months and less in Abu Dhabi, UAE.

Variable		Adjusted Odds Ratio (AOR)	(95% CI)	*p*-Value
Low maternal age, years		1.15	1.03, 1.30	0.018
Low gestational age, weeks		1.90	1.52, 2.36	<0.001
Nationality	No-Arab	0.35	0.07, 1.72	0.197
	Arab	Reference		
Received breastfeeding support	No	2.57	0.57, 11.63	0.222
	Yes	Reference		
Pre-pregnancy BMI	Abnormal	3.77	1.22, 11.76	0.022
	Normal	Reference		
Exclusive breastfeeding	No	1.90	0.49, 7.41	0.745
	Yes	Reference		
Initiation of breastfeeding	Delayed initiated	4.47	1.08, 18.48	0.012
	Timely initiated	Reference		

## References

[1] World Health Organization and UNICEF (2009). Baby-Friendly Hospital Initiative Revised, Updated and Expanded for Integrated Care.

[2] Kostuch M. (2019). New the Ten Steps to Successful Breastfeeding. Postępy Neonatologii.

[3] Centers for Disease Control and Prevention (2013). Breastfeeding Report Card.

[4] Cantey J., Bascik S., Heyne N., Gonzalez J., Jackson G., Rogers V., Sheffield J., Trevino S., Sendelbach D., Wendel G. (2012). Prevention of Mother-to-Infant Transmission of Influenza during the Postpartum Period. Am. J. Perinatol..

[5] Lee Y.M., Song K.H., Kim Y.M., Kang J.S., Chang J.Y., Seol H.J., Choi Y.S., Bae C.W. (2010). Complete rooming-in care of newborn infants. Korean J. Pediatr..

[6] Bystrova K., Matthiesen A.-S., Widström A.-M., Ransjo-Arvidson A.-B., Welles-Nystrom B., Vorontsov I., Uvnäs-Moberg K. (2007). The effect of Russian Maternity Home routines on breastfeeding and neonatal weight loss with special reference to swaddling. Early Hum. Dev..

[7] Ng C.A., Ho J.J., Lee Z.H. (2019). The effect of rooming-in on duration of breastfeeding: A systematic review of randomised and non-randomised prospective controlled studies. PLOS ONE.

[8] Pérez-Escamilla R., Martinez J.L., Segura-Pérez S. (2016). Impact of the Baby-friendly Hospital Initiative on breastfeeding and child health outcomes: A systematic review. Matern. Child Nutr..

[9] McRae M. (2019). Exclusive Breastfeeding, 24-Hour Rooming-In, and the Importance of Women’s Informed Choices. Nurs. Women’s Health.

[10] Tully K.P., Holditch-Davis D., Silva S., Brandon D. (2017). The Relationship Between Infant Feeding Outcomes and Maternal Emotional Well-being Among Mothers of Late Preterm and Term Infants. Adv. Neonatal Care.

[11] Declercq E.R., Sakala C., Corry M.P., Applebaum S. (2007). Listening to Mothers II: Report of the Second National, U.S. Survey of Women’s Childbearing Experiences: Conducted January-February 2006 for Childbirth Connection by Harris Interactive(R) in partnership with Lamaze. Int. J. Perin. Educ..

[12] Bystrova K., Widström A.-M., Matthiesen A.-S.T., Ransjö-Arvidson A.-B., Welles-Nyström B., Vorontsov I., Uvnäs-Moberg K. (2007). Early lactation performance in primiparous and multiparous women in relation to different maternity home practices. A randomised trial in St. Petersburg. Int. Breastfeed. J..

[13] Mikiel-Kostyra K., Mazur J., Wojdan-Godek E. (2005). Factors affecting exclusive breastfeeding in Poland: Cross-sectional survey of population-based samples. Int. J. Public Health.

[14] Radwan H. (2013). Patterns and determinants of breastfeeding and complementary feeding practices of Emirati Mothers in the United Arab Emirates. BMC Public Health.

[15] Azzeh F., Alazzeh A.Y., Hijazi H.H., Wazzan H.Y., Jawharji M.T., Jazar A., Filimban A.M., Alshamrani A.S., Labani M.S., Hasanain T.A. (2018). Factors Associated with Not Breastfeeding and Delaying the Early Initiation of Breastfeeding in Mecca Region, Saudi Arabia. Children.

[16] Bandeira de Sa N.N., Gubert M.B., Santos W.D., Santos L.M. (2016). Factors related to health services determine breastfeeding within one hour of birth in the Federal District of Brazil, 2011. Rev. Brasil. Epidemiol. Brazil. J. Epidemiol..

[17] Howe-Heyman A., Lutenbacher M. (2016). The Baby-Friendly Hospital Initiative as an Intervention to Improve Breastfeeding Rates: A Review of the Literature. J. Midwifery Women’s Health.

[18] Patterson J.A., Keuler N.S., Olson B. (2018). The effect of Baby-friendly status on exclusive breastfeeding in U.S. hospitals. Matern. Child Nutr..

[19] Taha Z. (2017). Trends of breastfeeding in the United Arab Emirates (UAE). Arab J. Nutr. Exerc..

[20] Taha Z., Garemo M., Nanda J. (2018). Patterns of breastfeeding practices among infants and young children in Abu Dhabi, United Arab Emirates. Int. Breastfeed. J..

[21] (2011). IBM SPSS Statistics for Windows Version 20.0.

[22] World Health Organization (2018). Global Database on Body Mass Index.

[23] Headen I., Cohen A., Mujahid M., Abrams B. (2017). The accuracy of self-reported pregnancy-related weight: A systematic review. Obes. Rev..

[24] El-Houfey A.A. (2017). Factors That Influence Exclusive Breastfeeding: A literature Review. Int. J. Nurs. Didact..

[25] Sipsma H.L., Jones K., Nickel N.C. (2017). Hospital practices to promote breastfeeding: The effect of maternal age. Birth.

[26] Santana G.S., Giugliani E.R.J., Vieira T.O., Vieira G.O. (2018). Factors associated with breastfeeding maintenance for 12 months or more: A systematic review. J. Pedi..

[27] Parker M.G., Ouyang F., Pearson C., Gillman M.W., Belfort M.B., Hong X., Wang G., Heffner L.J., Zuckerman B., Wang X. (2014). Prepregnancy body mass index and risk of preterm birth: Association heterogeneity by preterm subgroups. BMC Pregnancy Childbirth.

[28] Vinturache A.E., Moledina N., McDonald S., Slater D.M., Tough S. (2014). Pre-pregnancy Body Mass Index (BMI) and delivery outcomes in a Canadian population. BMC Pregnancy Childbirth.

[29] Kosa J.L., Guendelman S., Pearl M., Graham S., Abrams B., Kharrazi M. (2010). The Association Between Pre-pregnancy BMI and Preterm Delivery in a Diverse Southern California Population of Working Women. Matern. Child Heal. J..

[30] Alhaj A.M., Radi E.A., Adam I. (2010). Epidemiology of preterm birth in Omdurman Maternity hospital, Sudan. The journal of maternal-fetal & neonatal medicine: The official journal of the European Association of Perinatal Medicine, the Federation of Asia and Oceania Perinatal Societies. Int. Soc. Perinat. Obstet..

[31] Ladomenou F., Kafatos A., Galanakis E. (2007). Risk factors related to intention to breastfeed, early weaning and suboptimal duration of breastfeeding. Acta Paediatr..

[32] Tarrant R.C., Younger K.M., Sheridan-Pereira M., Kearney J. (2011). Factors Associated with Duration of Breastfeeding in Ireland. J. Hum. Lact..

[33] Consales A., Crippa B.L., Cerasani J., Morniroli D., Damonte M., Bettinelli M.E., Consonni D., Colombo L., Zanotta L., Bezze E. (2020). Overcoming Rooming-In Barriers: A Survey on Mothers’ Perspectives. Front. Pediatr..

[34] Mikiel-Kostyra K., Mazur J., Bołtruszko I. (2002). Effect of early skin-to-skin contact after delivery on duration of breastfeeding: A prospective cohort study. Acta Paediatr..

[35] McGrath S.K., Jh K. (2002). Extended mother-infant skin-to-skin contact and prospect of breastfeeding. Acta Paediatr..

[36] Breslow N.E., Day N.E. (1980). Statistical methods in cancer research. Volume I—The analysis of case-control studies. IARC Sci. Publ..

[37] Li R., Scanlon K.S., Serdula M.K. (2005). The validity and reliability of maternal recall of breastfeeding practice. Nutrit. Rev..

[38] Cattaneo A., DaVanzo R., Ronfani L. (2000). Are data on the prevalence and duration of breastfeeding reliable? The case of Italy. Acta Paediatr..

